# Editorial: Break the stigma: autism

**DOI:** 10.3389/fpsyt.2024.1513447

**Published:** 2024-12-04

**Authors:** Nichole E. Scheerer, Catalina Sau Man Ng, Ava N. Gurba, Morgan L. McNair, Matthew D. Lerner, April Hargreaves

**Affiliations:** ^1^ Department of Psychology, Wilfrid Laurier University, Waterloo, ON, Canada; ^2^ Department of Early Childhood Education, The Education University of Hong Kong, Hong Kong, Hong Kong SAR, China; ^3^ AJ Drexel Autism Institute, Drexel University, Philadelphia, PA, United States; ^4^ Department of Psychology, Stony Brook University, Stony Brook, NY, United States; ^5^ Department of Psychology, National College of Ireland, Dublin, Ireland

**Keywords:** stigma, autism, integration, isolation, neurodiversity

In this Research Topic we bring together a collection of research to *Break the Stigma* associated with autism, by exploring the impact of autism stigma and discussing the ways in which we can combat stigma.

Autistic individuals, transgender/gender non-conforming (TGNC) autistic people (Glaves and Kolman) and by extension, their caregivers (Clarke et al.) often experience victimization, bullying, and stigmatization. The pervasiveness of this stigma is evident when reviewing interviews of young autistic adults (Marion et al.) who all experienced stigma in the form of exclusion or isolation, with many also experiencing verbal bullying. However, this stigma is not reserved for autistic people. Twenty caregivers of profoundly autistic adults reported that they experienced at least one perceived stigma, characterized by negative responses or interactions with people in the community (Clarke et al.). These stigmatizing interactions were also reported with educators, peers (Marion et al.) and clinicians (Glaves and Kolman).

Stigma can be extremely impactful. For autistic people, stigma increases camouflaging behaviours aimed at concealing their autistic traits (Rivera and Bennetto), it interferes with their development of self-determination and autonomy (Thompson-Hodgetts et al.), it undermines their psychosocial well-being (Glaves and Kolman; Marion et al.), and it leads to adverse consequences such as suicidality (i.e., suicidal ideation, self-harm and suicidal attempts (Shaw et al.). Further, for parents of autistic children, stigma around bilingualism was shown to discourage families from raising their autistic child bilingually (Digard et al.).

Autism stigma has been also shown to significantly impede the integration of autistic individuals into society, a theme addressed by multiple studies in this special edition. Persistent barriers, inadequate support systems, and entrenched societal attitudes exacerbate this issue, as is often seen within education. For example, in South Korea, Yoon et al. highlight how systemic stigmatization in secondary education leads to bullying, trauma, and exclusion from further education and employment. The study emphasizes how societal values like elitism and meritocracy worsen these challenges, underscoring the need for targeted interventions.

This issue is not unique to Korea. Ahlers et al. explored the isolation of autistic students in self-contained classrooms in the U.S. and revealed that educators’ attitudes contribute to this exclusion. They advocate for more inclusive practices that extend beyond physical integration and call for strategies to enhance educators’ understanding of autism. One such strategy, investigated by Jenks et al. in the UK, is to run a training program for university staff aimed at debunking stereotypes and improving understanding of autism. Although quantitative measures showed limited changes, qualitative data revealed substantial benefits, particularly through including autistic perspectives. This approach enhanced staff’s nuanced understanding and practical application in their interactions with autistic students.

Beyond education, integration barriers also exist in social service provision. Li and Qi examined the challenges faced by NGOs in China working with autistic children, noting how funding structures and interactions with funders impacted the effectiveness of inclusion efforts. This study provides valuable insights into the barriers in social service provision that hinder the integration of autistic children and suggests the need for more targeted and effective solutions.

In broader society, Boucher et al. explored how non-autistic adults quickly form negative judgments about autistic children based on brief interactions. They found that adults with higher social competence and explicit autism stigma were more likely to hold negative perceptions of autistic children. Similarly, Jones and Sasson found that college undergraduates often displayed patronizing and exclusionary attitudes towards autism. These studies underscore the need to address biases that contribute to social exclusion.

Collectively, these studies call for more focus on autistic strengths, inclusive practices, better-informed societal attitudes, and targeted interventions to support the integration of autistic individuals across various educational and social contexts. From initial diagnosis, clinicians should provide strength-based information to highlight autism strengths and reduce stigma (Woods and Estes). To provide more support to autistic people, Shaw et al. suggest a neurodiversity-affirmative approach to autism which may promote a more positive self-identity and improved mental health. Similarly, Riebel et al. highlight the role of promoting self-compassion in reducing the self-stigma and shame often associated with autism. Researchers also emphasized the need to provide more opportunities for autistic people to make choices and exert autonomy (Thompson-Hodgetts et al.; McVey et al.). For example, Glaves and Kolman advise that clinicians should take an intersectional perspective of their autistic clients’ gender identities to reduce stigma and have a better understanding of the needs of the whole person. Providing adequate support and better educating autistic medical professionals may promote inclusion in the medical workforce (Shaw et al.).

Another salient theme in this Research Topic was the role of research(ers) in perpetuating autism stigma. As researchers, we need to explicitly address the link between ableism and poor autism science (Bottema-Beutel et al.). This means shifting away from research that reduces autistic people to their perceived deficits and instead focuses on how socially constructed views of “abilities” contribute to autistic people’s “disabilities”. This can be achieved by centering autistic voices (Kaplan-Khan and Caplan; McVey et al.; Caldwell-Harris et al.). *Comprehensive participatory research* promotes close collaboration with the autistic community and other autism stakeholders across all stages of research, allowing autistic people to share their perspectives and shape research priorities (Bottema-Beutel et al.; Kaplan-Khan and Caplan; McVey et al.). Researchers also need to leverage the unique contributions autistic researchers bring to autism research. For example, when qualitative interviews are conducted by autistic/non-autistic researcher dyads, autistic participants report increased connection and comfort (Kaplan-Khan and Caplan). Approaches like these that centre the autistic voice facilitate closer alignment and trust between autism research(ers) and the autistic community, promote novel research programs that are relevant to the priorities of autistic people, create more ethical and less ableist research practices, and ultimately culminate in reduced autism stigma.

Together the articles in this Research Topic stress the fact that researchers, clinicians, and society more broadly need to do a better job at advocated for the rights of autistic people. This starts with the understanding that autistic people are a marginalized population that experience discrimination (McVey et al.). It is not autism itself that leads to a poor quality of life, but instead, a lack of social support and acceptance. Social interactions are bidirectional, yet autistic people are under enormous pressure to learn about and accommodate the needs and preferences of non-autistic people (Schuck and Fung). We need to shift our focus away from the outdated notion that autistic people need to be fixed and instead place the onus on non-autistic people to learn about and accommodate the needs of autistic people (McVey et al.). By teaching and promoting neurodiversity, or the understanding that there are no “right” kinds of brains, non-autistic people can learn to accept and value autistic differences (Jenks et al.; Schuck and Fung; Davidson and Morales). Virtual autism acceptance programs for children (Davidson and Morales) and high schoolers (Schuck and Fung), and a training program for higher education staff (Jenks et al.), all demonstrated success in reducing stigma, but stigma reduction can also be achieved through greater social inclusion. For example, service dog placements were found to act as a social catalyst, decreasing experiences of judgement and stigma for autistic children by inviting others to approach and interact (Leighton et al.). By increasing non-autistic people’s acceptance of individuals whose behaviours may not align with society’s expectations, autistic people may garner more social support and ultimately experience an improvement in their quality of life.

